# Esophageal Submucosal Giant Hematoma Detected After Mitral Repair Using Transesophageal Echocardiography

**DOI:** 10.7759/cureus.27292

**Published:** 2022-07-26

**Authors:** Keiji Akao, Yusuke Ishida, Koichi Nakazawa, Toshio Okada, Toshiki Fujiyoshi, Aya Kawachi, Hiroyuki Uchino

**Affiliations:** 1 Department of Anesthesiology, Tokyo Medical University Hospital, Tokyo, JPN; 2 Department of Cardiovascular Surgery, Tokyo Medical University Hospital, Tokyo, JPN

**Keywords:** cardiopulmonary bypass, anticoagulant, esophageal stricture, giant esophageal submucosal hematoma, transesophageal echocardiography (tee)

## Abstract

Transesophageal echocardiography (TEE) is a necessary diagnostic tool for cardiac surgery, including for intraoperative evaluation of the morphology and function of each structure. On the other hand, many complications caused by insertion and manipulation of the TEE probe have been reported, such as gastrointestinal injuries and hematoma, as well as esophageal perforation. Here, we report a case in which a large submucosal esophageal hematoma was found on the fourth postoperative day after surgery using TEE for mitral regurgitation. The patient was an 81-year-old man who underwent mitral valve replacement for mitral regurgitation. On the fourth postoperative day, anorexia and blood-tinged sputum were observed. A computed tomography (CT) scan of the chest displayed a giant esophageal submucosal hematoma. When performing TEE, to avoid complications, it is important to handle the TEE probe with care and to avoid leaving the device at the same site for long periods of time.

## Introduction

Since its introduction in the operating room in the early 1980s, TEE has become widely used during cardiac, macrovascular, and other surgical procedures. TEE is an essential tool for the intraoperative evaluation of cardiac morphology and function and position verification associated with surgical manipulation. However, many complications associated with TEE probe insertion and manipulation have been reported. Here, we report a case of a patient with mitral valve regurgitation who developed a postoperative giant esophageal submucosal hematoma after the mitral valve replacement, a TEE-guided cardiac surgical procedure. We obtained written informed consent from the patient to publish this case report.

## Case presentation

The patient was an 81-year-old man (height: 157 cm; weight: 48 kg; body mass index {BMI}: 19.5 kg/m^2^). He was American Society of Anesthesiologists (ASA) physical class III. He had been diagnosed and treated for mitral valve regurgitation and tricuspid valve regurgitation eight years prior to this surgery. An electrocardiogram showed atrial fibrillation about 6 months previously, and the mitral valve regurgitation was worsening. Therefore, the patient was scheduled for mitral valve replacement, tricuspid valvuloplasty, and maze procedure under general anesthesia. He had a history of benign prostatic hypertrophy, which was being treated with silodosin. The patient was taking oral edoxaban (30 mg/day) as an anticoagulant for thrombotic risk owing to atrial fibrillation. The oral anticoagulation was continued until the day before surgery.

**Figure 1:**
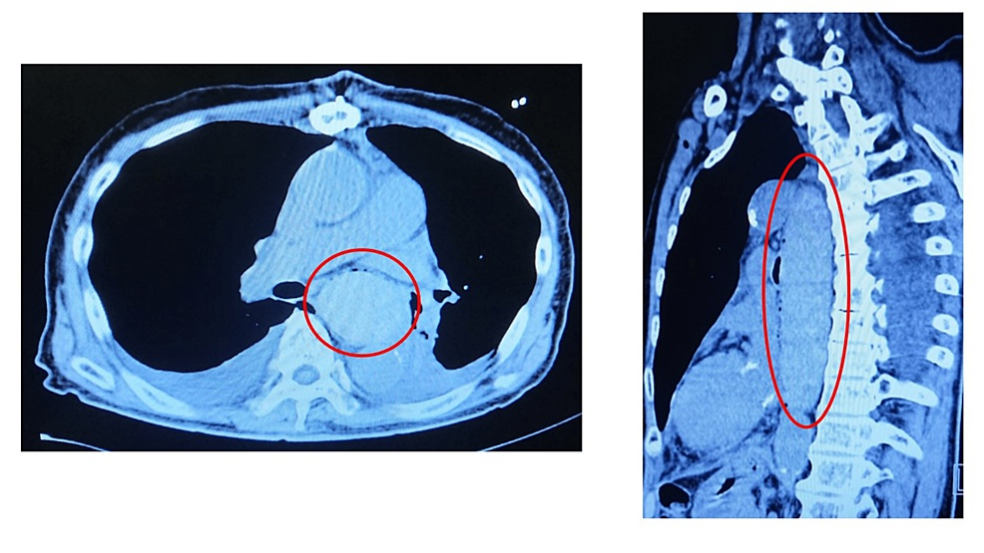
Chest CT images, axial (left) and sagittal (right) views, taken on postoperative day 3. The images show a giant esophageal submucosal hematoma (45×50×210 mm). CT: computed tomography

Insertion of a TEE probe (ACUSON SC2000 PRIME; Erlangen, Germany: SIEMENS Healthineers) at the time of anesthesia induction was performed without any problems. Mitral valve replacement (bioprosthetic valve), tricuspid valvuloplasty, and maze procedure were performed as scheduled under cardiopulmonary bypass. After weaning from cardiopulmonary bypass, circulation dynamics were stable, and the operation was completed. The heart and aorta had been observed by the intraoperative TEE as needed. However, there had been no findings in the esophagus that suggested a hematoma. The durations of the surgery, anesthesia, and cardiopulmonary bypass were 369, 452, and 192 min, respectively. The estimated blood loss was 1,919 mL. The patient was transfused with packed red cells (1,400 mL), fresh frozen plasma (480 mL), and autologous blood (400 mL). His preoperative hemoglobin level was 12.2 g/dL and postoperative hemoglobin level was 10.8 g/dL. There was a little blood noted on the TEE probe when it was removed. The patient was transferred to the intensive care unit under sedation with mechanical ventilation and was extubated on the same day. On postoperative day (POD) 2, the patient started a fluid diet. However, the patient experienced a loss of appetite and demonstrated blood in the sputum. An otolaryngological examination did not show any hemorrhage in the pharynx and larynx regions. Chest CT examination revealed a giant hematoma in the esophageal submucous region and esophageal stenosis (Figure 1). The hematoma was limited to the submucosal region, and no esophageal perforation was found. Therefore, the patient was conservatively managed by avoiding food intake and he received total parenteral nutrition infusion.

**Figure 2:**
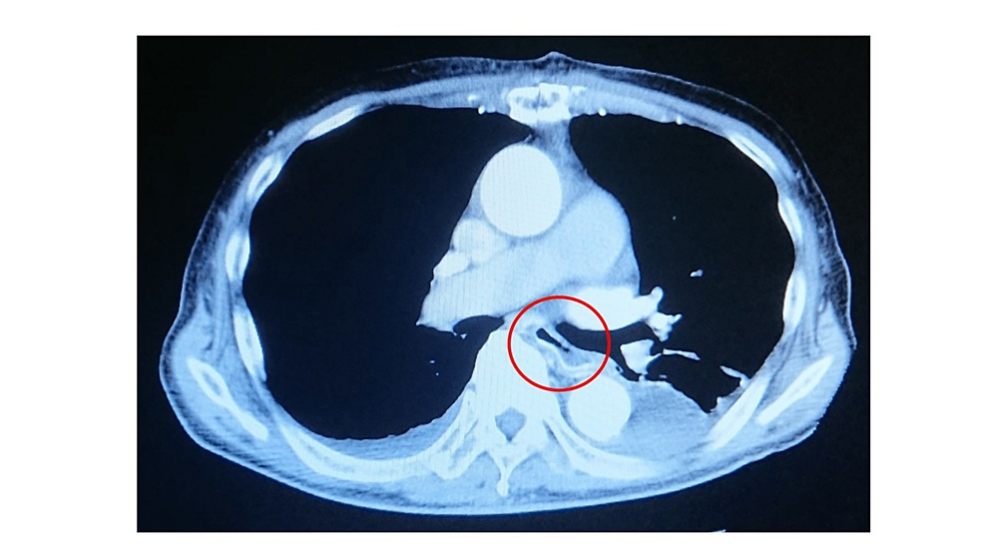
Chest CT image, axial view, taken on postoperative day 11. The giant submucosal esophageal hematoma has resolved. CT: computed tomography

**Figure 3:**
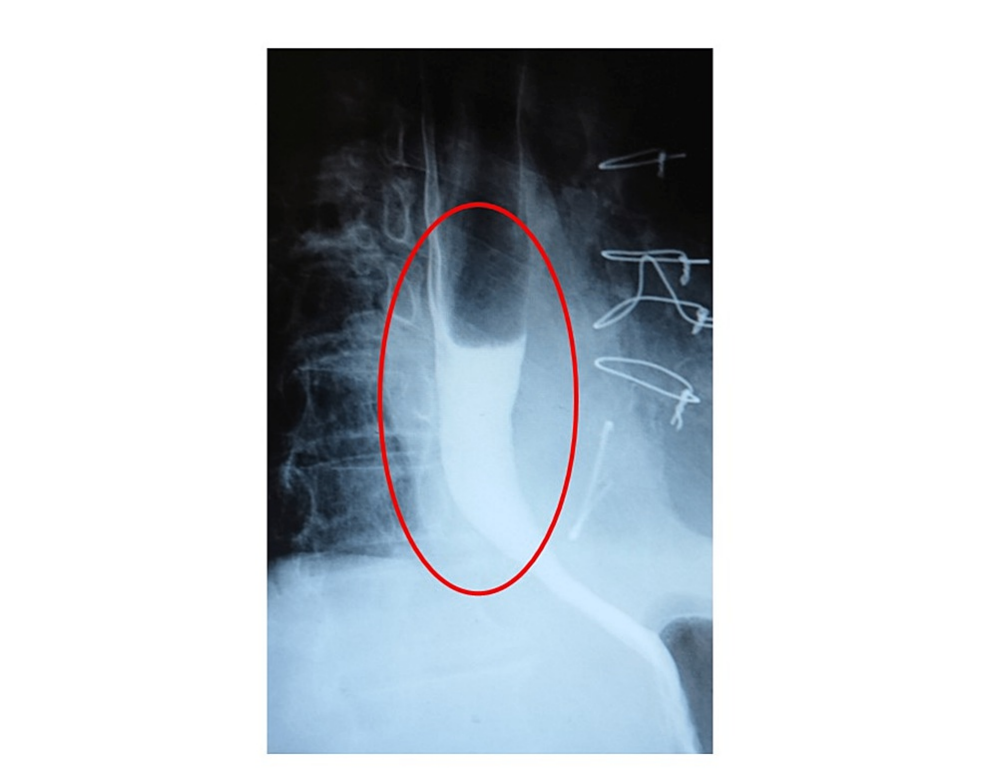
Upper gastrointestinal tract radiography performed on postoperative day 26. The image shows no signs of esophageal transit disorder.

Despite the giant hematoma, we continued to treat the patient with anticoagulants. No bloody sputum was observed on POD 6, and chest CT on POD 7 confirmed that the hematoma had shrunk (Figure 2). Esophageal fluoroscopy on POD 26 confirmed no stenosis (Figure 3). The patient started a liquid diet again from POD 27, and his subsequent clinical course was favorable. The patient was discharged from the hospital on POD 41.

## Discussion

The intraoperative TEE procedure is associated with mechanical damage caused by ultrasound probe insertion. It has been known that swallowing disturbance, tooth dislodgment, dislocation of the tracheal tube, and digestive tract bleeding and/or perforation are rarely seen following the TEE procedure [1,2]. The incidence rate of such complications has been reported to be 1% and less, and the rate of serious complications, such as digestive tract perforation, has been reported to be 0.02-0.04% [3,4]. Risk factors of TEE complications include old age, a low BMI, anatomical abnormalities, surgery except for coronary artery bypass graft surgery, cerebrovascular events, long cardiopulmonary bypass time, etc. [5]. Causes of digestive tract damage include mucosal ischemia by mechanical stimulation of the TEE probe, heat damage, and low tissue perfusion during cardiopulmonary bypass, and these are all possible causes of hematoma formation in the present patient [6]. Although mucosal damage by the TEE probe can be detected during the surgery if the hemodynamic condition is changed, the damage is often detected several days after the surgery, similar to the present case.

In the present case, the shape of the probe tip was rounded, which was unlikely to have caused the injury. But ultrasound probe manipulation was frequently required during the surgery, owing to continuous monitoring during mitral valve replacement and tricuspid valvuloplasty, resulting in esophageal injury. On the other hand, TEE-induced esophageal hematoma often results from a small mucosal tear followed by “aggressive” heparin anticoagulation as intraoperative and postoperative anticoagulant therapy. The risk of digestive tract injury should always be kept in mind when TEE is used.

It is necessary to manipulate the ultrasound probe gently, avoid placement of the probe at the same place for a long time, and hold the probe at the resting position when it is not being used. In the current case, the intraoperative ultrasound image hadn’t shown abnormal findings. However, if intraoperative ultrasound image had shown abnormal findings, further evaluations including CT examination at the earliest time point after the surgery might have enabled us to earlier diagnose [7].

A large esophageal hematoma may compress the heart and cause hemodynamic instability [8,9]. The airway may also be compressed, resulting in respiratory distress [10]. Furthermore, esophageal hematomas can cause substantial morbidity owing to a prolonged hospital stay, potential for residual esophageal strictures, and dysmotility in the long term [11]. Therefore, it is important to avoid causing esophageal hematomas. In addition, if an esophageal hematoma develops, early recognition and detection can help improve hospital stay and prevent further complications [12]. In general, esophageal hematomas should be treated conservatively by analgesia, cessation of anticoagulation, and cessation of oral intake [11,12]. However, in some patients, anticoagulation must be continued because they had undergone valve replacement. In addition, proton-pump inhibitors were also prescribed in this case because of their potential to treat esophageal hematomas and reduce the risk of gastrointestinal bleeding during anticoagulation therapy [13].

## Conclusions

Here, we reported a case of a patient who developed a giant submucosal hematoma potentially owing to TEE probe manipulation. Therefore, it is necessary to be cognizant of the types and causes of complications that can occur at the time of insertion and during manipulation of the TEE probe, and special attention should be paid to prevent such complications.

## References

[1] Hilberath JN, Oakes DA, Shernan SK, Bulwer BE, D'Ambra MN, Eltzschig HK (2010). Safety of transesophageal echocardiography. J Am Soc Echocardiogr.

[2] Kallmeyer IJ, Collard CD, Fox JA, Body SC, Shernan SK (2001). The safety of intraoperative transesophageal echocardiography: a case series of 7200 cardiac surgical patients. Anesth Analg.

[3] Min JK, Spencer KT, Furlong KT, DeCara JM, Sugeng L, Ward RP, Lang RM (2005). Clinical features of complications from transesophageal echocardiography: a single-center case series of 10,000 consecutive examinations. J Am Soc Echocardiogr.

[4] Augoustides JG, Hosalkar HH, Milas BL, Acker M, Savino JS (2006). Upper gastrointestinal injuries related to perioperative transesophageal echocardiography: index case, literature review, classification proposal, and call for a registry. J Cardiothorac Vasc Anesth.

[5] Patel KM, Desai RG, Trivedi K, Neuburger PJ, Krishnan S, Potestio CP (2022). Complications of transesophageal echocardiography: a review of injuries, risk factors, and management. J Cardiothorac Vasc Anesth.

[6] Purza R, Ghosh S, Walker C, Hiebert B, Koley L, Mackenzie GS, Grocott HP (2017). Transesophageal echocardiography complications in adult cardiac surgery: a retrospective cohort study. Ann Thorac Surg.

[7] Sharma B, Lowe D, Antoine M, Shah M, Szyjkowski R (2019). Intramural esophageal hematoma secondary to food ingestion. Cureus.

[8] Nault I, Bertrand OF (2007). Severe haemodynamic compromise due to left atrial compression by oesophageal haematoma. Heart.

[9] Modi P, Edwards A, Fox B, Rahamim J (2005). Dissecting intramural haematoma of the oesophagus. Eur J Cardiothorac Surg.

[10] McCall R, Thomas SP (2009). Esophageal hematoma complicating catheter ablation for atrial fibrillation. J Cardiovasc Electrophysiol.

[11] Kumar S, Ling LH, Halloran K (2012). Esophageal hematoma after atrial fibrillation ablation: incidence, clinical features, and sequelae of esophageal injury of a different sort. Circ Arrhythm Electrophysiol.

[12] Singh D, Zaheer K, Dobariya V, Lester AP, Teka S (2020). The conundrum of treating portal vein thrombosis and submucosal esophageal hematoma. Cureus.

[13] Dong Y, He S, Li X, Zhou Z (2022). Prevention of nnon-vitamin K oral anticoagulants-related gastrointestinal bleeding with acid suppressants: a systematic review and meta-analysis. Clin Appl Thromb Hemost.

